# Immunohistochemical localisation of vasopressin/oxytocin-type, corazonin-type and luqin-type neuropeptide expression in the starfish *Asterias rubens* using antibodies to the C-terminal region of precursor proteins

**DOI:** 10.1007/s00441-023-03738-w

**Published:** 2023-01-19

**Authors:** Ana B. Tinoco, Michaela Egertová, Maurice R. Elphick

**Affiliations:** School of Biological & Behavioural Sciences, Queen Mary University of London, Mile End Road, London, E1 4NS UK

**Keywords:** Neuropeptide, Vasopressin/oxytocin, Corazonin, Luqin, Echinoderm

## Abstract

Neuropeptides derived from larger precursor proteins are secreted as signalling molecules by neurons and regulate diverse physiological and behavioural processes in animals. Recently, we reported the discovery of ArCRZ (HNTFTMGGQNRWKAG-NH_2_) and ArLQ (EEKTRFPKFMRW-NH_2_)—novel neuropeptides in the starfish *Asterias rubens* that are orthologs of arthropod corazonins and molluscan luqins, respectively. However, our efforts to generate antibodies to ArCRZ and ArLQ have been unsuccessful, precluding immunohistochemical analysis of their expression. Here, we investigated an alternative experimental approach for neuropeptide immunohistochemistry by generating antibodies to peptides corresponding to the C-terminal region of the precursor proteins. As proof of principle, we generated antibodies to the C-terminal region of the precursor of the vasopressin/oxytocin-type neuropeptide asterotocin and show that these reveal immunostaining in *A. rubens* that is very similar to that observed with asterotocin antibodies. Furthermore, antibodies to the C-terminal region of the ArCRZ precursor (ArCRZP) and the ArLQ precursor (ArLQP) produced patterns of immunostaining consistent, respectively, with the distribution of ArCRZP and ArLQP transcripts revealed by mRNA in situ hybridisation. Detailed immunohistochemical analysis revealed widespread expression of ArCRZP and ArLQP in *A. rubens*, including the central nervous system, digestive system and the body wall and its associated appendages (e.g. tube feet), providing a neuroanatomical framework for investigation and interpretation of the pharmacological actions of ArCRZ and ArLQ in *A. rubens*. Furthermore, our findings provide a basis for use of antibodies to the C-terminal region of neuropeptide precursor proteins in other species where the production of antibodies to the bioactive neuropeptides is unsuccessful.

## Introduction

Neuropeptides are secreted neuronal signalling molecules derived from larger precursor proteins that typically exert effects by binding to specific G-protein coupled receptors. A huge variety of neuropeptides have been discovered in animals, and these are involved in the regulation of diverse physiological and behavioural processes (Jekely et al. 2018; Dockray 1987; Elphick et al. 2018). Furthermore, the evolutionary origin of at least thirty neuropeptide types can be traced back to the urbilaterian common ancestor of protostomes and deuterostomes (Jekely 2013; Mirabeau and Joly 2013; Elphick et al. 2018), including corazonin-type, luqin-type and vasopressin/oxytocin-type neuropeptides that are the focus of this study.

The neuropeptide corazonin was first isolated from the cockroach *Periplaneta americana* on account of its cardioexcitatory effects, with the name “corazonin” being derived from the Spanish word “corazon”, which means “heart” (Veenstra 1989). Identification of the receptor that mediates the effects of corazonin in the fruit-fly *Drosophila melanogaster* revealed a close relationship with the adipokinetic hormone (AKH) signalling system in insects and the gonadotropin-releasing hormone (GnRH) signalling system in vertebrates (Cazzamali et al. 2002; Park et al. 2002). Furthermore, it was proposed that corazonin-type signalling may be restricted in its phylogenetic distribution to the protostome branch of the Bilateria (Hauser and Grimmelikhuijzen 2014). However, our discovery that the neuropeptide HNTFTMGGQNRWKAG-NH_2_ (ArCRZ) acts as a ligand for a corazonin-type receptor (ArCRZR) in a deuterostome, the starfish *A. rubens*, revealed that the evolutionary origin of corazonin-type signalling can be traced back further to the common ancestor of the Bilateria (Tian et al. 2016; Zandawala et al. 2018). The discovery of ArCRZ in *A. rubens* also enabled the first investigation of the expression and pharmacological actions of a corazonin-type neuropeptide in a deuterostome (Tian et al. 2017). Thus, it was discovered that ArCRZ is a myoexcitatory neuropeptide that causes contraction of in vitro preparations of the apical muscle, the cardiac stomach and tube feet from *A. rubens*. Consistent with these actions, expression of ArCRZ precursor transcripts was revealed in the apical muscle, cardiac stomach and tube feet of *A. rubens* using mRNA in situ hybridisation methods. However, the investigation of ArCRZ expression in *A. rubens* using immunohistochemical methods was thwarted by failed attempts to generate specific antibodies to ArCRZ (Tian et al. 2017). This contrasts with our investigations of many other neuropeptides in *A. rubens*, where the generation of neuropeptide-specific antibodies has been successful and has facilitated detailed immunohistochemical analysis of neuropeptide expression (Cai et al. 2018, 2021; Lin et al. 2017, 2018; Tian et al. 2017; Zhang et al. 2020, 2022; Odekunle et al. 2019; Tinoco et al. 2021). Thus, one of the aims of this study was to circumvent the failure to generate antibodies to ArCRZ by instead producing antibodies to the ArCRZ precursor protein.

The neuropeptide luqin was discovered and named on account of its expression by neurons located in the left upper quadrant of the abdominal ganglion in the mollusc *Aplysia californica* (Aloyz and DesGroseillers 1995; Shyamala et al. 1986; Yañez-Guerra and Elphick 2020). Luqin-type neuropeptides were also discovered in other molluscs such as the snail *Achatina fullica*, revealing a physiological role as cardioexcitatory peptides (Fujimoto et al. 1990). Furthermore, the G-protein coupled receptor for a luqin-type neuropeptide was discovered in the pond snail *Lymnaea stagnalis* (Tensen et al. 1998). More recently, and with the availability of transcriptome/genome sequence data from an ever-growing variety of taxa, the phylogenetic distribution of luqin-type signalling has been investigated. This has revealed that the luqin-type signalling system in molluscs and other lophotrochozoan protostomes is orthologous to the ecdysozoan RYamide-type neuropeptide signalling system (Jekely 2013; Mirabeau and Joly 2013), which has been functionally characterised in insects and the nematode *Caenorhabditis elegans* (Ohno et al. 2017; Yañez-Guerra and Elphick 2020). Furthermore, luqin-type neuropeptide precursors and receptors were also identified in non-chordate deuterostomes such as the echinoderm *Strongylocentrotus purpuratus* (sea urchin) and the hemichordate *Saccoglossus kowalevskii* (acorn worm) (Jekely 2013; Mirabeau and Joly 2013). Thus, it has been inferred that the evolutionary origin of luqin-type neuropeptide signalling can be traced back to the common ancestor of the Bilateria, but with the loss of this signalling system having occurred in the chordate lineage (Yañez-Guerra and Elphick 2020; Yañez-Guerra et al. 2018). Furthermore, the discovery of luqin-type neuropeptides in ambulacrarians (echinoderms and hemichordates) has enabled the first functional characterisation of luqin-type signalling in deuterostomes. Thus, having identified a transcript encoding a luqin-type precursor in the starfish *A. rubens* (ArLQP; (Semmens et al. 2016)), we recently reported a detailed characterisation of the luqin-type signalling system in this species (Yañez-Guerra et al. 2018), and subsequently, the luqin-type signalling system has also been characterised in another echinoderm species—the sea cucumber *Apostichopus japonicus* (Li et al. 2022). Our mass spectrometric analysis of *A. rubens* radial nerve cord extracts enabled the determination of the mature structure of the luqin-type peptide derived from ArLQP—EEKTRFPKFMRW-NH_2_ (ArLQ). Furthermore, two G-protein coupled receptors (ArLQR1 and ArLQR2) were identified experimentally as receptors for ArLQ in *A. rubens*. Investigation of the in vitro pharmacological actions of ArLQ on organ preparations from *A. rubens* revealed that it caused dose-dependent relaxation of tube feet but it had no effect on the cardiac stomach. Consistent with the effect of ArLQ on tube feet, expression of ArLQP transcripts was revealed in tube feet using mRNA in situ hybridisation methods (Yañez-Guerra et al. 2018). However, as with ArCRZ (see above), we have thus far been unsuccessful in generating antibodies to ArLQ that could be used for immunohistochemical analysis of its expression in *A. rubens* (Yañez-Guerra 2018).

The aim of this study was to investigate if antibodies to neuropeptide precursor proteins can be used as an alternative approach for the visualisation of neuropeptide expression in *A. rubens*. This approach to the visualisation of neuropeptide expression has been reported in other taxa; for example, antibodies to a non-neuropeptide fragment of the somatostatin precursor have been used for immunohistochemical analysis of expression in the rat retina (Larsen et al. 1990; Larsen 1995). To assess the validity of this experimental approach in *A. rubens*, we first generated antibodies to the precursor of the vasopressin/oxytocin-type neuropeptide asterotocin to enable immunohistochemical comparison with antibodies to asterotocin, which were generated previously as part of a comprehensive functional characterisation of vasopressin/oxytocin-type neuropeptide signalling in *A. rubens* (Odekunle et al. 2019). Importantly, antibodies to the C-terminal region of the asterotocin precursor revealed a pattern of immunostaining in *A. rubens* that was strikingly similar to that obtained with asterotocin antibodies, demonstrating the validity of this experimental approach. Informed by these findings, we then generated antibodies to peptides corresponding to the C-terminal region of the ArCRZ and ArLQ precursors and employed these antibodies for immunohistochemical analysis of their expression in *A. rubens*.

## Materials and methods

### Animals

Specimens of starfish (*Asterias rubens*) with a diameter > 4 cm were collected at low tide from the Thanet Coast (Kent, UK) or were obtained from a fisherman based at Whitstable (Kent, UK). The starfish were maintained in an aquarium with circulating seawater (salinity of 32 ‰) under a 12 h–12 h light–dark cycle (lights on at 8 a.m.) at a temperature of ~12 °C, located in the School of Biological & Behavioural Sciences at Queen Mary University of London. Animals were fed on mussels (*Mytilus edulis*) that were collected at low tide near Margate (Kent, UK). Additionally, juvenile specimens of *A. rubens* (diameter 0.5–1.5 cm) were collected from the University of Gothenburg Sven Lovén Centre for Marine Infrastructure (Kristineberg, Sweden).

### Generation and characterisation of antibodies to ArASTP, ArCRZP and ArLQP

**Fig. 1:**
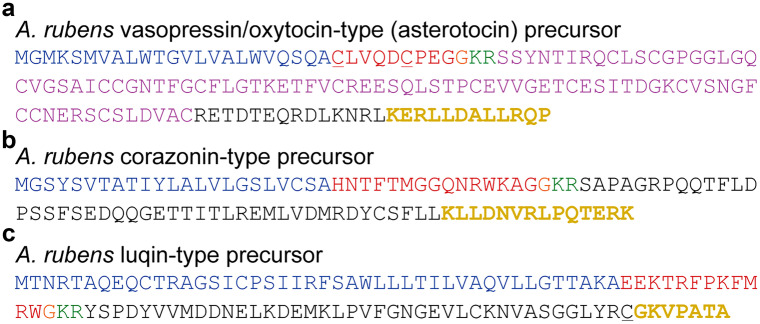
Amino acid sequences of **a** vasopressin/oxytocin-type (asterotocin) precursor (ArASTP), **b** corazonin-type precursor (ArCRZP) and **c** luqin-type precursor (ArLQP) in *Asterias rubens*. Predicted signal peptides are shown in blue, neuropeptides are shown in red but with C-terminal glycine residues that are substrates for amidation shown in orange, dibasic cleavage sites are shown in green and the neurophysin domain of ArASTP is shown in pink. The cysteine residues in ArASTP that form a disulphide bridge in the mature neuropeptide are underlined. The sequences of the C-terminal peptides that were used as antigens for antibody production are shown in bold yellow. Note, however, that the underlined cysteine residue in ArLQP was replaced with a lysine residue at the N-terminus of the antigen peptide to provide reactive sites for glutaraldehyde-mediated coupling to a carrier protein (thyroglobulin). GenBank Accession numbers: **a** ALJ99953.1 (Semmens et al. 2016; Odekunle et al. 2019); **b** ALJ99955.1 (Semmens et al. 2016; Tian et al. 2017, 2016); **c** ALJ99961.1 (Semmens et al. 2016; Yañez-Guerra et al. 2018)

Peptides corresponding to the C-terminal region of the *A. rubens* asterotocin precursor (ArASTP; KERLLDALLRQP; Fig. 1a), corazonin-type precursor (ArCRZP; KLLDNVRLPQTERK; Fig. 1b) and luqin-type precursor (ArLQP; KGKVPATA; Fig. 1c) were custom synthesized by Peptide Protein Research Ltd (Fareham, UK). Naturally occurring lysine residues at the N-terminus of ArASTP and ArCRZP peptide antigens and an introduced lysine at the N-terminus of the ArLQP peptide antigen, replacing a naturally occurring cysteine residue (Fig. 1c), facilitated glutaraldehyde-mediated coupling to porcine thyroglobulin (Sigma-Aldrich, Gillingham, UK) as a carrier protein, using 5% glutaraldehyde (Sigma-Aldrich, Gillingham, UK) in phosphate buffer (0.1 M; pH 7.2). Then, antigen peptide-thyroglobulin conjugates were used for the immunisation of one rabbit per antigen peptide (70-day protocol; Charles River Biologics, Romans, France). Conjugates were emulsified in Freund’s complete adjuvant for primary immunisations (~100 nmol antigen peptide) and in Freund’s incomplete adjuvant for three booster immunisations (~50 nmol antigen peptide). The presence of antibodies to the antigen peptides in post-immunisation serum samples was assessed using an enzyme-linked immunosorbent assay (ELISA; see below), in comparison with pre-immune serum.

Antibodies to the antigen peptides were purified from the final bleed antiserum by affinity purification using the AminoLink Plus Immobilization Kit (Thermo Fisher Scientific, Waltham, MA), with bound antibodies eluted using glycine elution buffer (6.3 ml of 100 mM glycine [VWR Chemicals, Leicestershire, UK] and 0.7 ml of Tris [1 M, pH = 7.0]) and trimethylamine (TEA) elution buffer (6.3 ml of TEA [Sigma-Aldrich, Gillingham, UK] and 0.7 ml of Tris [1 M, pH = 7.0]). Eluates were dialysed and sodium azide (0.1%) was added for long-term storage of the affinity-purified antibodies at 4 °C. ArASTP, ArCRZP and ArLQP antibodies eluted with TEA were diluted in 5% normal goat serum (NGS; Sigma-Aldrich, Gillingham, UK)/PBST (phosphate-buffered saline containing 0.1% Tween-20) at 1:20, 1:15 and 1:15, respectively, and then used for immunohistochemistry (see below). The rabbit antisera to ArASTP, ArCRZP and ArLQP have been assigned the RRID numbers RRID:AB_2922389, RRID:AB_2922390 and RRID:AB_2922391, respectively.

### Enzyme-linked immunosorbent assay (ELISA)

To assess the production of antibodies during the immunization protocol and following collection of a terminal bleed, enzyme-linked immunosorbent assays (ELISA) were carried out to test antisera for the presence of antibodies to ArASTP, ArCRZP and ArLQP. One hundred microliters of a 1 μM solution of antigen peptide were added to each well of a PVC microtitre plate (Starlab, Milton Keynes, UK) overnight at 4 °C. After washing with PBS, 200 μl 5% NGS was added to each well at room temperature for 2 h to block non-specific binding sites. Then after washing with PBST, varying dilutions of antiserum or pre-immune serum (1:500 to 1:16,000 diluted in 5% NGS/PBS) were added to each well. After overnight incubation at 4 °C, the antiserum solutions were washed away and each well was incubated with alkaline phosphatase (AP)-labelled goat anti-rabbit IgG secondary antibodies (1:3000 in 5% NGS/PBS [Thermo Fisher Scientific, Oxford, UK]) for 3 h at room temperature. Then after washing with PBST, p-nitrophenylphosphate alkaline phosphatase substrate (Vector Laboratories, Peterborough, UK) was added to each well for a 20 min incubation at room temperature. Lastly, absorbance at 415 nm was measured using a FLUOstar Omega plate reader (BMG LABTECH, Ortenberg, Germany) and then the mean absorbance values were calculated and plotted using Prism 6.

### Immunohistochemical localisation of ArASTP, ArCRZP and ArLQP in *A. rubens*

Specimens of *A. rubens* were fixed by immersion in seawater Bouin’s fluid (75% saturated picric acid [Sigma-Aldrich, Gillingham, UK] in seawater, 25% formaldehyde, 5% acetic acid) for 3–4 days at 4 °C and then were decalcified for a week using a 2% ascorbic acid/0.3 M sodium chloride solution. Following dehydration and embedding in paraffin wax, sections of arms and the central disk region (8–10 μm; transverse or horizontal) were cut using a microtome (RM 2145, Leica Microsystems [UK], Milton Keynes, UK) and mounted on chrome alum/gelatin-coated microscope slides. Paraffin wax was removed by immersion of slides in xylene, and then slides were immersed in 100% ethanol. Endogenous peroxidase activity was quenched using a 0.3% hydrogen peroxide (VWT Chemicals, Leicestershire, UK)/methanol solution for 30 min. Subsequently, the slides were rehydrated through a graded ethanol series (90%, 70%, and 50%) and distilled water, blocked in 5% NGS made up in PBST.

Following preliminary tests in which antisera were tested at a range of concentrations, the specificity of immunostaining was assessed by testing antisera alongside antisera pre-absorbed with the corresponding antigen peptide. For these experiments, the ArASTP, ArCRZP and ArLQP antisera were tested at dilutions of 1:4000, 1:1000 and 1:4000 in PBS, respectively. For pre-absorption, the ArASTP, ArCRZP and ArLQP antisera were first prepared at dilutions of 1:400, 1:100 and 1:400 in PBS, respectively, and then were incubated with the corresponding antigen peptide at a concentration of 200 µM for 2 h at room temperature. Then, the pre-absorbed antisera were further diluted 1:10 in 5% NGS/PBST so that they were tested on starfish sections at the same final concentrations as the antisera without pre-absorption. After overnight incubation of slides with antisera or pre-absorbed antisera followed by a series of washes in PBST, indirect immunohistochemical detection was carried out using Peroxidase-AffiniPure Goat Anti-Rabbit IgG (H + L) conjugated to Horseradish Peroxidase (RRID: AB_2313567; Jackson ImmunoResearch, West Grove, PA) diluted 1:1000 in 2% NGS/PBST. Bound antibodies were revealed using a solution containing 0.015% hydrogen peroxide, 0.05% diaminobenzidine (VWR Chemicals, Leicestershire, UK) and 0.05% nickel chloride (Sigma-Aldrich, Gillingham, UK) in PBS. When strong staining was observed, sections were washed in distilled water, dehydrated through a graded ethanol series (50%, 70%, 90% and 100%) and washed in xylene before being mounted with coverslips on DPX mounting medium (Thermo Fisher Scientific, Waltham, MA).

Having investigated the specificity of immunostaining with antisera, a more extensive immunohistochemical analysis was performed using the same methods as described above, but employing the use of affinity-purified antibodies to ArASTP, ArLQP and ArCRZP (TEA fraction diluted 1:20, 1:15 and 1:15, respectively, in 5% NGS/PBST). For experiments with ArASTP antibodies, adjacent sections were incubated with affinity-purified rabbit antibodies to asterotocin (TEA fraction diluted 1:15 in 5% NGS/PBST), which have been reported previously (Odekunle et al. 2019).

### Imaging

**Fig. 2:**
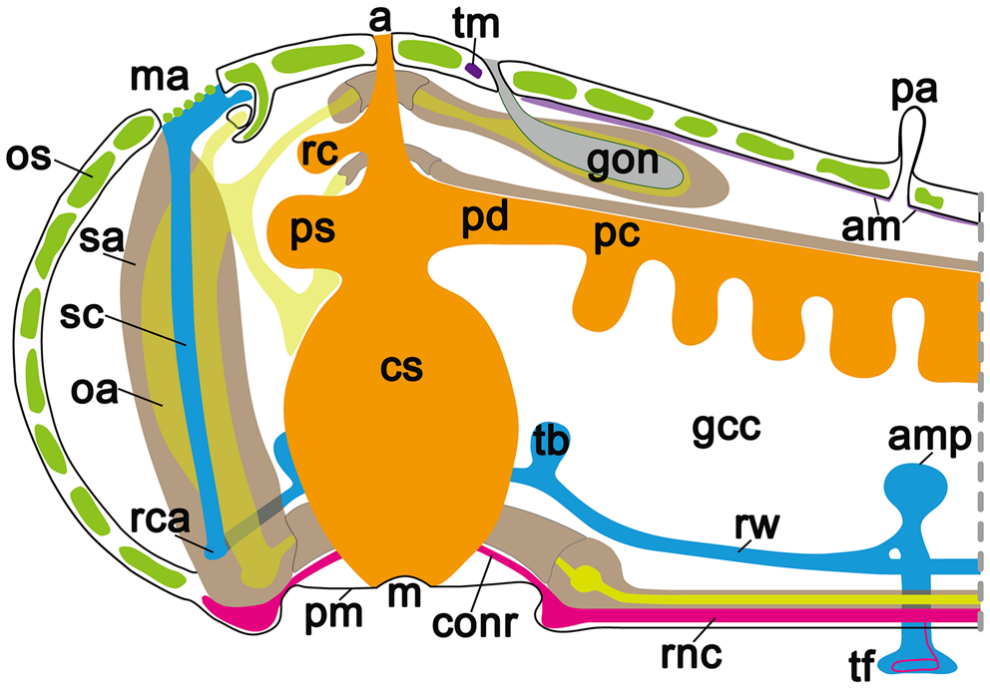
Graphical representation of starfish anatomy showing a vertical section of the central disk and the proximal region of an adjoining arm. Colour key: body wall skeleton, green; digestive system, orange; hemal system, brown; muscles, purple; nervous system, pink; perihemal system, yellow; reproductive system, grey; water vascular system, blue. Abbreviations: a, anus; amp, ampulla; am, apical muscle; cs, cardiac stomach; conr, circumoral nerve ring; gcc, general coelomic cavity; gon, gonad; m, mouth; ma, madreporite; oa, organ axial; os, ossicle; pa, papullae; pm, peristomial membrane; pc, pyloric caecum; pd, pyloric duct; ps, pyloric stomach; rc, rectal caecum; rnc, radial nerve cord; rw, radial water vascular canal; sa, sinus of axial organ; sc, stone canal; tm, tourniquet muscle; tb, Tiedemann’s body; tf, tube foot. Diagram was modified from Yañez-Guerra et al. (2018)

Photographs of sections processed for immunohistochemistry were captured using a INFINITY5-5C Color Camera (Teledyne Lumenera, Ontario, CA) linked to a DMRA2 light microscope (Leica), utilising INFINITY ANALYZE v.7.0.2.920 image analysis software (Teledyne Lumenera, Ontario, CA) running on an iMac computer (27-inch, Late 2013 model with OS X Yosemite, version 10.10). Montages of photographs were prepared using Adobe Photoshop CC (version 19.1.4, × 64) and Adobe Illustrator CC (version 22.1, × 64) running on a MacBook Pro computer (13-inch, early 2015 model with OS Monterrey version 12.2.1). Interpretation of the patterns of immunostaining reported here can be made with reference to Fig. 2, which shows a graphic representation of starfish anatomy.

## Results

### Comparison of immunostaining in *A. rubens* obtained using antibodies to asterotocin and antibodies to the asterotocin precursor

**Fig. 3:**
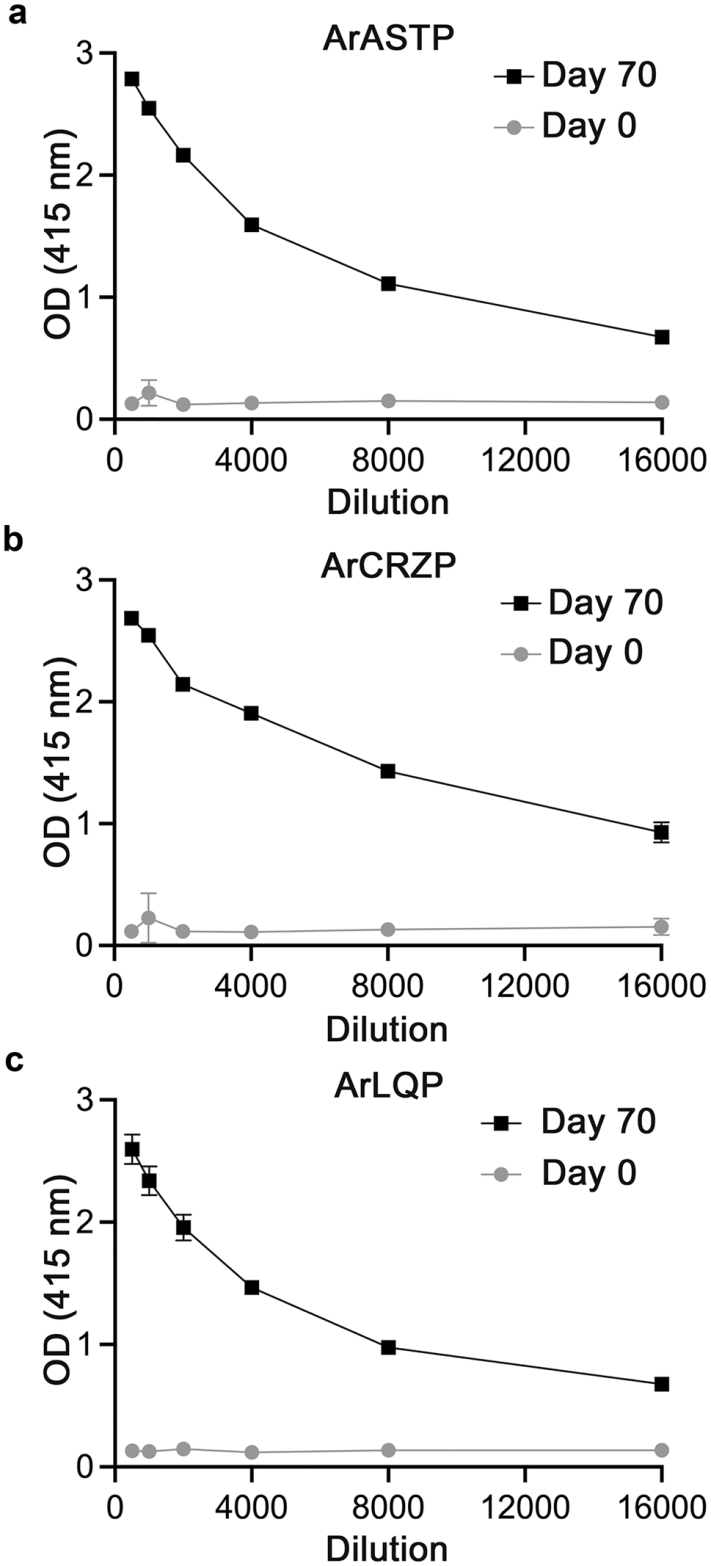
Characterisation of rabbit antibodies to ArASTP, ArCRZP and ArLQP using an enzyme-linked immunosorbent assay (ELISA). **a** 0.1 nmol of the ArASTP antigen peptide is detected by the ArASTP antiserum (black line) at dilutions between 1:500 and 1:16,000, with the presence and titre of antibodies to the ArASTP antigen peptide being inferred by comparison with pre-immune serum (grey line). **b** 0.1 nmol of the ArCRZP antigen peptide is detected by the ArCRZP antiserum (black line) at dilutions between 1:500 and 1:16,000, with the presence and titre of antibodies to the ArCRZP antigen peptide being inferred by comparison with pre-immune serum (grey line). **c** 0.1 nmol of the ArLQP antigen peptide is detected by the ArLQP antiserum (black line) at dilutions between 1:500 and 1:16,000, with the presence and titre of antibodies to the ArLQP antigen peptide being inferred by comparison with pre-immune serum (grey line). All data points are mean values from three replicates

**Fig. 4:**
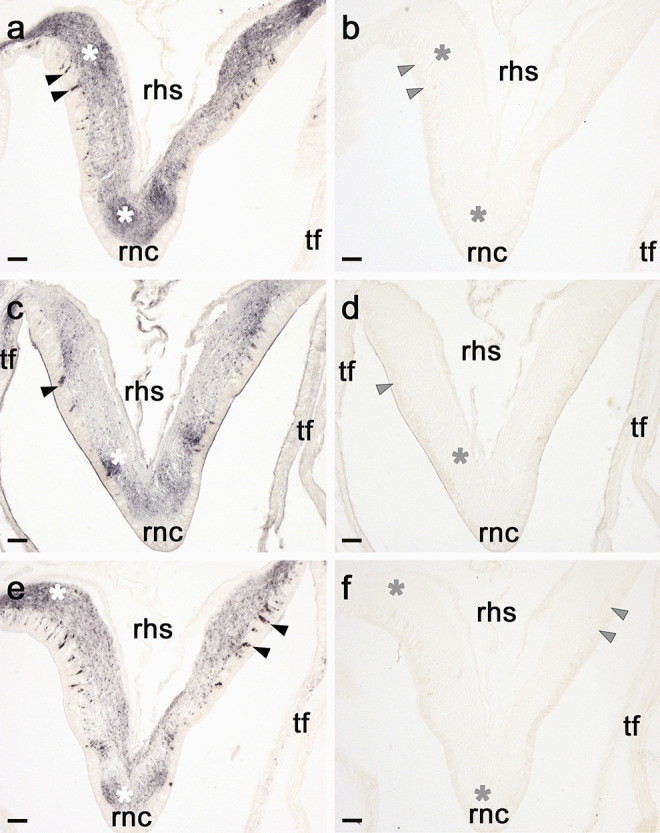
Immunostaining in the radial nerve cord of *A. rubens* observed with neuropeptide precursor antisera is abolished by pre-absorption of antisera with antigen peptides. **a** ArASTP antiserum, **b** ArASTP antiserum pre-absorbed with antigen peptide, **c** ArACRZP antiserum, **d** ArCRZP antiserum pre-absorbed with antigen peptide, **e** ArLQP antiserum, **f** ArLQP antiserum pre-absorbed with antigen peptide. Immunostaining can be seen in transverse sections of the V-shaped radial nerve cords, with regions of the ectoneural neuropile containing a high density of immunostained fibres (white asterisks in **a**, **c**, **e**), while these (grey asterisks) and other regions of the neuropile are void of staining in adjacent sections in **b**, **d**, **f**. Selected immunostained cell bodies are labelled with black arrowheads in **a**, **c** and **e**, whilst in adjacent sections, there is an absence of immunostained cell bodies in these (grey arrowheads) and other regions of the ectoneural epithelium in **b**, **d**, **f**. Abbreviations: rhs, radial hemal strand; rnc, radial nerve cord; tf, tube foot. Scale bars: a—f = 25 μm

**Fig. 5:**
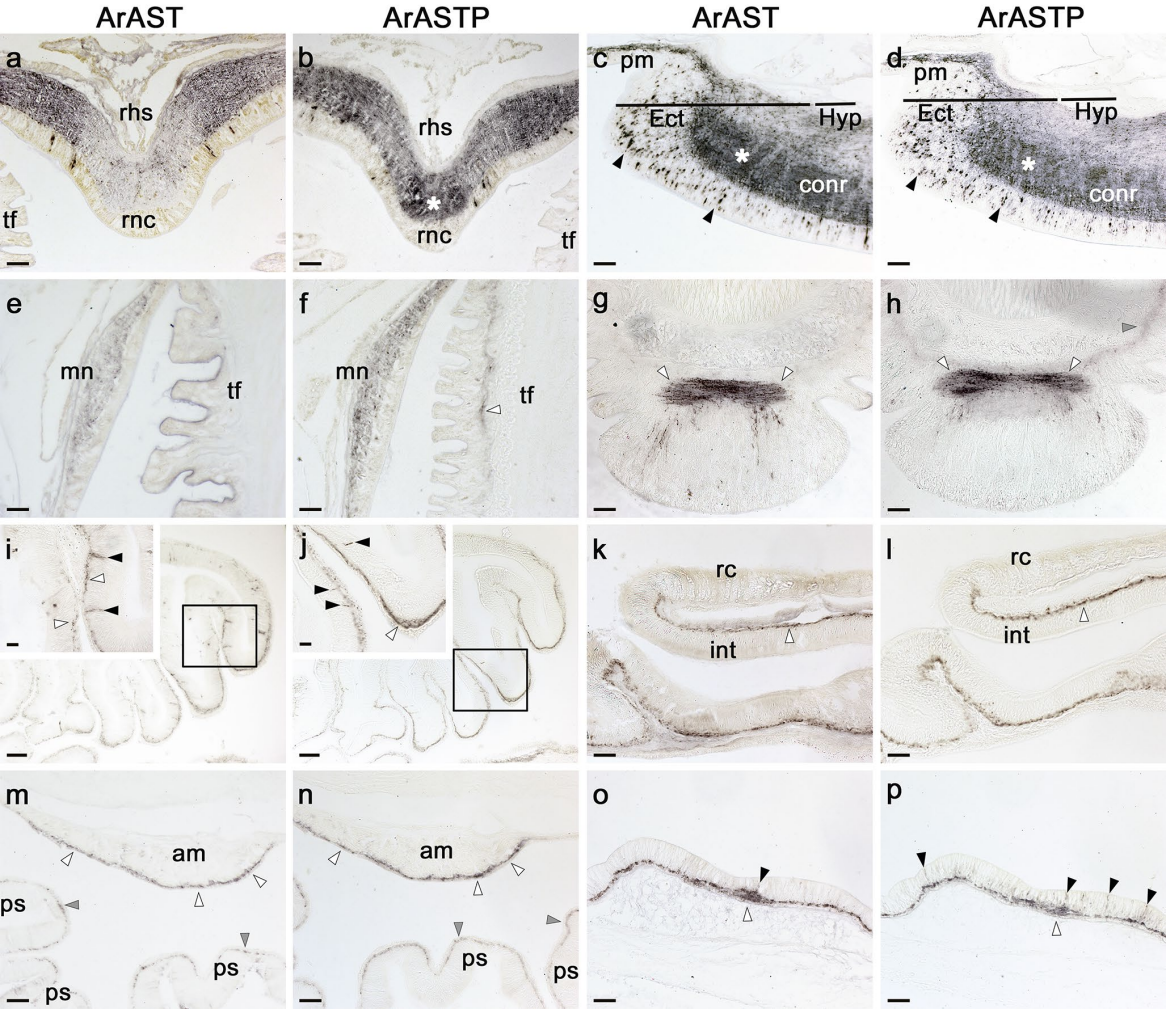
Comparative immunohistochemical analysis of vasopressin/oxytocin-type neuropeptide expression in the starfish *Asterias rubens* using antibodies to asterotocin (ArAST) and antibodies to the C-terminal region of the asterotocin precursor (ArASTP). In different regions of the starfish body shown here, similar patterns of immunostaining are observed in adjacent sections labelled with ArAST antibodies or ArASTP antibodies. **a**, **b** Radial nerve cord (V-shaped in transverse section); the patterns of immunostaining are similar, with the exception of the apical region of the nerve cord, which exhibits ArASTP-immunoreactivity (ArASTP-ir; *) but is largely void of ArAST-immunoreactivity (ArAST-ir). **c**, **d** Circumoral nerve ring; immunostained cells can be seen in the epithelium of the ectoneural region (arrowheads) and a region of the ectoneural neuropile containing a higher density of immunostained fibres is highlighted with a white asterisk. **e**, **f** Marginal nerve and adjacent tube foot; ArASTP-ir, but not ArAST-ir, is revealed in the subepithelial nerve plexus of the tube foot (arrowhead). **g**, **h** Disk region of a tube foot, showing ArAST-ir and ArASTP-ir in the basal nerve ring (white arrowheads), whereas in the subepithelial nerve plexus only ArASTP-ir is detected (grey arrowhead). **i**, **j** Lateral pouches of the cardiac stomach, with insets showing boxed regions at higher magnification. Immunostaining is observed in cells located in the mucosal epithelium (black arrowheads) and in the basiepithelial nerve plexus (white arrowheads). **k**, **l** Intestine. Immunostaining is present in the basiepithelial nerve plexus of the intestine (white arrowheads), but the rectal caecum is void of immunostaining. **m**, **n** Apical muscle and pyloric stomach. Immunostaining is present in the basiepithelial nerve plexus located beneath the coelomic epithelium of the apical muscle (white arrowheads) and in the basiepithelial nerve plexus of the pyloric stomach (grey arrowheads). **o**, **p** Body wall. Immunostaining is present in cells located in the external epithelium (black arrowheads) and in the subepithelial nerve plexus (white arrowhead). Abbreviations: am, apical muscle; conr, circumoral nerve ring; Ect, ectoneural; Hyp, hyponeural; int, intestine; mn, marginal nerve; pm, peristomial membrane; ps, pyloric stomach; rc, rectal caecum; rhs, radial hemal strand; rnc, radial nerve cord; tf, tube foot. Scale bars: i, j = 60 μm; a, b, c, d, g, h, k, l, m, n, o, *p* = 32 μm; e, f, i-inset, j-inset = 16 μm

Enzyme-linked immunosorbent assay (ELISA) analysis of serum from a rabbit immunized with an antigen peptide comprising the C-terminal region of the *A. rubens* asterotocin precursor (ArASTP) revealed the presence of antibodies to the antigen peptide (Fig. 3a). Immunohistochemical tests in which the ArASTP antiserum was tested alongside ArASTP antiserum pre-absorbed with the antigen peptide revealed the specificity of immunostaining observed in the radial nerve cord (Fig. 4a,b). However, to optimise immunolabelling, affinity-purified antibodies were used for detailed analysis of ArASTP expression in *A. rubens*. To accomplish this, ArASTP antigen-specific antibodies were affinity-purified (TEA eluate; 1:20 dilution) and used alongside affinity-purified rabbit asterotocin antibodies (TEA eluate; 1:15; (Odekunle et al. 2019)) for immunohistochemical tests on adjacent sections of arms and central disks from *A. rubens*. Importantly, the patterns of immunostaining obtained with the two antibodies were very similar, as described in detail below and as illustrated in Fig. 5.

The central nervous system (CNS) of *A. rubens* comprises radial nerve cords that extend along the oral side of each arm and which are linked by a circumoral nerve ring in the central disk (Fig. 2). Two rows of tube feet are located on each side of the radial nerve cords, and a single row of tube feet is located around the circumoral nerve ring. Running parallel with the radial nerve cords are smaller marginal nerves, which are located lateral to the outer row of tube feet on each side of the arm. A specialized tube foot-like organ located at the tips of each arm is the terminal tentacle, a sensory organ with a photosensitive and pigmented optic cushion located at its base (Smith 1937; Mashanov et al. 2016). The overall pattern of immunostaining obtained with both antibodies in the CNS was similar, with immunostained cells located in the ectoneural epithelium and extensive immunostaining of fibres in the underlying neuropile region and no immunostaining in the hyponeural region (Fig. 5a–d). Consistent regional differences in the density or intensity of immunostaining in the ectoneural neuropile were observed with both antibodies, and this is most clearly seen in the circumoral nerve ring in Fig. 5c, d. Intense staining in the lateral regions of the radial nerve cord ectoneural neuropile is also a consistent feature of both antibodies (Fig. 5a, b). However, a difference in the pattern of immunostaining was observed in the apical region of the V-shaped radial nerve cords, with weak/diffuse immunostaining observed with asterotocin antibodies but with dense/intense immunostaining observed with ArASTP antibodies (Fig. 5a, b). Similar patterns of immunostaining are observed with both antibodies in the marginal nerve cords (Fig. 5e, f). In the tube feet (Fig. 2), the ArASTP antibodies revealed immunostaining in the subepithelial nerve plexus that was not detected in adjacent sections with asterotocin antibodies (Fig. 5e, f, g, h). However, similarly, intense immunostaining was observed in the basal nerve ring above the tube foot disk region with both antibodies (Fig. 5g, h).

The digestive system of *A. rubens* comprises a mouth, located on the underside of the central disk, surrounded by a contractile peristomial membrane, which is continuous aborally with a short tubular oesophagus. The stomach comprises two compartments: a large and highly folded cardiac stomach, which is everted through the mouth during feeding, and a smaller pyloric stomach, which is linked via a short intestine to the rectum, rectal caeca and anus. Paired digestive glands or pyloric caeca are located in each arm, and these are connected to the pyloric stomach by pyloric ducts (Fig. 2) (Jangoux 1982). Similar patterns of immunostaining were obtained with both antibodies in the digestive system. For example, in the lateral pouches of the cardiac stomach, immunostained cells in the mucosal layer and immunostained fibres in the basiepithelial nerve plexus are revealed with both antibodies (Fig. 5i, j). Likewise, immunostained fibres in the basiepithelial nerve plexus of the intestine (Fig. 5k, l) and pyloric stomach (Fig. 5m–n) are revealed by both antibodies.

The body wall skeleton of *A. rubens* comprises calcite ossicles (Fig. 2) that are interconnected by muscles and collagenous tissue. The external surface of the body wall has a variety of appendages that include protective pincer-like pedicellariae and spines as well as thin-walled gas exchange organs known as papulae (Fig. 2). The internal surface of the body wall is lined by a coelomic epithelium, which is underlain by longitudinally and circularly orientated muscle layers. Along the midline of each arm, the longitudinal muscle layer of the coelomic lining is thickened to form the apical muscle (Fig. 2), which facilitates flexion of the arm (Blowes et al. 2017). Similar patterns of immunostaining were observed with both antibodies in the body wall. For example, in the nerve plexus underlying the coelomic epithelium of the apical muscle (Fig. 5m, n), in cells located in the external epithelium (Fig. 5o, p) and in fibres in the subepithelial nerve plexus underlying the external epithelium of the body wall (Fig. 5o, p).

#### Immunohistochemical localization of ArCRZP in *A. rubens*

ELISA analysis of serum from a rabbit immunized with an antigen peptide comprising the C-terminal region of the *A. rubens* corazonin-type precursor (ArCRZP) revealed the presence of antibodies to the antigen peptide (Fig. 3b). Immunohistochemical tests in which the ArCRZP antiserum was tested alongside ArCRZP antiserum pre-absorbed with the antigen peptide revealed specificity of immunostaining observed in the radial nerve cord (Fig. 4c, d). However, to optimise immunolabelling, affinity-purified antibodies (TEA eluate; 1:15 dilution) were used for detailed immunohistochemical analysis of ArCRZP expression in *A. rubens*.

**Fig. 6:**
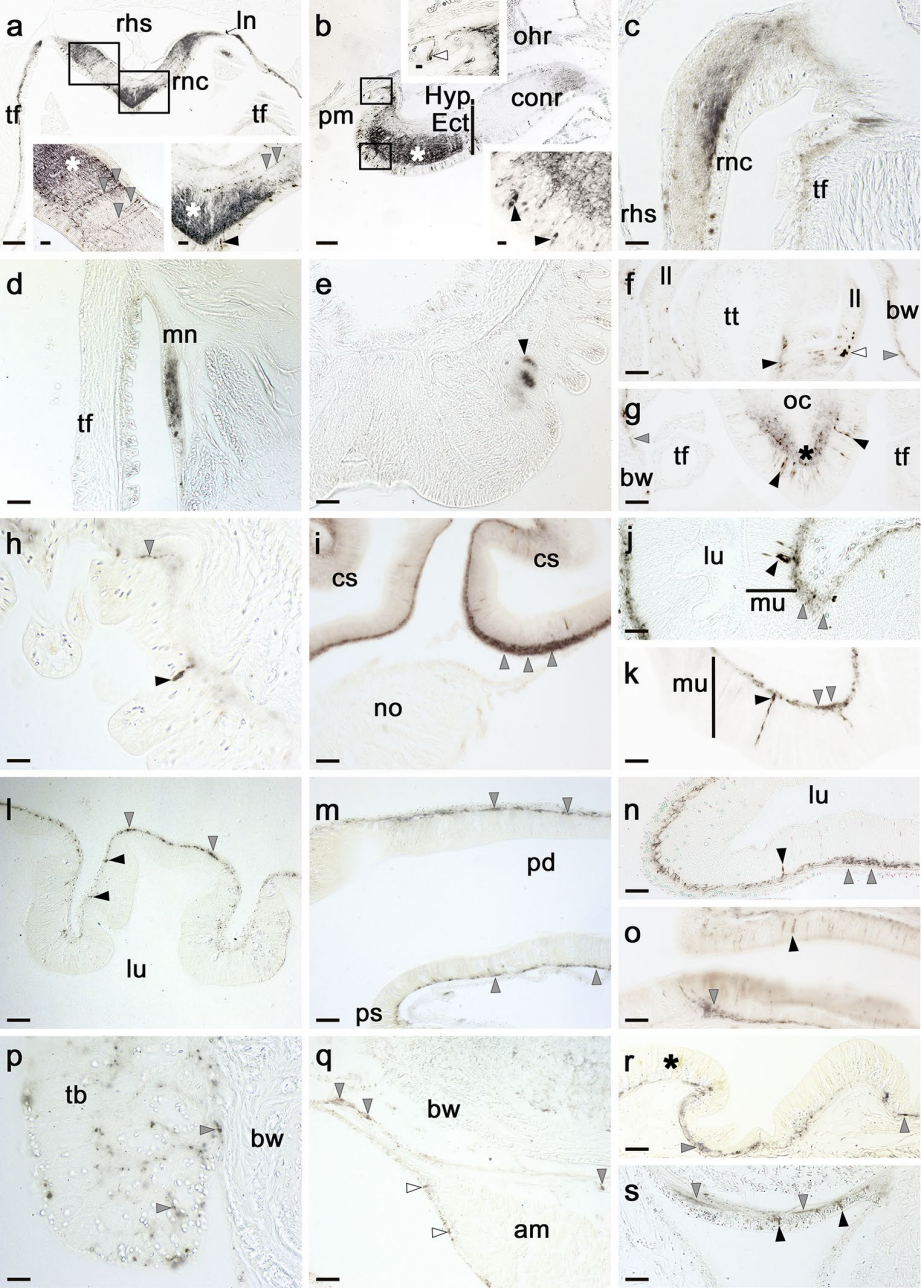
Imunohistochemical localisation of ArCRZP in *Asterias rubens*. **a** ArCRZP-immunoreactivity (ArCRZP-ir) in a transverse section of the V-shaped radial nerve cord, with bilaterally symmetrical regional variation in the density of immunostaining in the ectoneural neuropile. The boxed regions are at higher magnification in the insets, with regions of the ectoneural neuropile containing a high density of immunostained fibres labelled with asterisks. Immunostained cell bodies can be seen in the ectoneural epithelium (black arrowheads) and punctate immunostained fibres can be seen projecting across the ectoneural neuropile in different orientations (grey arrowheads). Immunostaining can also be seen in a lateral nerve emanating from the radial nerve cord and in the subepithelial nerve plexus of an adjacent tube foot. **b** ArCRZP-ir in the circumoral nerve ring, showing regional variation in the density of immunostaining in the ectoneural neuropile; the boxed regions are at higher magnification in the insets, showing immunostained cells in the ectoneural epithelium (black arrowheads) and in the external epithelial layer of the peristomial membrane (white arrowhead) near to the junction with the circumoral nerve ring. **c** High magnification image of the junction between the radial nerve cord and an adjacent tube foot, showing ArCRZP-ir in the ectoneural neuropile of the radial nerve cord and in the subepithelial nerve plexus of the adjacent tube foot. **d** ArCRZP-ir in the marginal nerve. **e** ArCRZP-ir in the basal nerve ring (black arrowheads) of a tube foot. **f** Horizontal section of an arm tip showing ArCRZP-ir in the terminal tentacle (black arrowhead), lateral lappet (white arrowhead) and subepithelial nerve plexus of the body wall surrounding the terminal tentacle (grey arrowhead). **g** Horizontal section of an arm tip showing ArCRZP-ir in bipolar-shaped cells (black arrowheads) located in the photosensory epithelium and in the underlying neuropile (*) of the optic cushion. **h** High magnification transverse section of the oesophagus showing an immunostained cell (black arrowhead) in the mucosal layer and immunostained processes (grey arrowhead) in the basiepithelial nerve plexus. **i** ArCRZP-ir in the lateral pouches of the cardiac stomach; note that the density of immunostained fibres is highest (grey arrowheads) in regions of the basiepithelial nerve plexus adjacent to the retractor strand and nodule, which are unstained. **j**, **k** High magnification images of cardiac stomach tissue showing ArCRZP-ir in cell bodies (black arrowheads) and in the basiepithelial nerve plexus (grey arrowheads). **l** ArCRZP-ir in the pyloric stomach showing immunostaining in cells (black arrowheads) of the mucosa layer and in the basiepithelial nerve plexus (grey arrowhead). **m** Longitudinal section of an arm showing ArCRZP-ir in the basiepithelial nerve plexus of a pyloric duct (grey arrowheads). **n** ArCRZP-ir in a cell body (black arrowhead) and in the basiepithelial nerve plexus (grey arrowheads) of a pyloric caecum. **o** ArCRZP-ir in the intestine, with immunostaining located in cells of the mucosal layer (black arrowhead) and in the basiepithelial nerve plexus (grey arrowheads). **p** Immunostained processes (grey arrowheads) in a Tiedemann’s body, which is a specialised organ involved in the regulation of the composition of the coelomic fluid. **q** ArCRZP-ir located beneath the coelomic epithelium of an apical muscle (white arrowheads) and in the circular muscle layer of the coelomic lining of the body wall (grey arrowheads). **r**, **s** ArCRZP-ir in the subepithelial nerve plexus of the external epithelium of the body wall (grey arrowheads) on both aboral (**r**) and oral (**s**) sides of the body. On the oral side, the stained cells (black arrowheads) can be seen to be located between the spines of the body wall. On the aboral side of the body wall, the external epithelium is naturally pigmented, and this is labelled with an asterisk to highlight that this is not ArCRZP-ir. Abbreviations: am, apical muscle; bnp, basiepithelial nerve plexus; bw, body wall; conr, circumoral nerve ring; cs, cardiac stomach; Ect, ectoneural; Hyp, hyponeural; int, intestine; ln, lateral nerve; ll, lateral lappet; lu, lumen; mn, marginal nerve; mu, mucosa; no, nodule; oc, optic cushion; ohr, oral hemal ring; pd, pyloric duct; pm, peristomial membrane; ps, pyloric stomach; rhs, radial hemal strand; rnc, radial nerve cord; tb, Tiedeman’s body; tf, tube foot; tt, terminal tentacle. Scale bars: a = 120 μm; b = 60 μm; d, e, f, g, i, l, m, n, o, q, r, s = 32 μm; a-insets, c, h, j, k, p = 16 μm; b-insets = 6 μm

Antibodies to ArCRZP revealed immunostaining in the major components of the nervous system of *A. rubens*, including the radial nerve cords, the circumoral nerve ring and the marginal nerves (Fig. 6a–d). Analysis of staining in the radial nerve cord and circumoral nerve ring revealed immunostained in cells in the ectoneural region but not in the hyponeural region. The immunostained cells in the ectoneural region were distributed throughout the sub-cuticular epithelial layer (Fig. 6a, b). Furthermore, a dense network of immunostained fibres was revealed in the ectoneural neuropile, with bilaterally symmetrical regional variation in the density of immunostaining (Fig. 6a, b); for example, dense and intense immunostaining is observed in the lateral and apical regions of the ectoneural neuropile in the radial nerve cords, whereas in the intervening neuropile only a few sparsely distributed, but intensely stained, fibres are present. Immunostained cells and/or processes were also revealed in the lateral branches of the radial nerve cords (Fig. 6a), in the subepithelial nerve plexus of the tube feet (Fig. 6a, c) and in the marginal nerves (Fig. 6d). In the tube feet, immunostaining was also were revealed in the basal nerve ring of the disk region (Fig. 6e). The terminal tentacle is a specialised tube foot-like sensory organ located at the tips of each of the arms in starfish. Immunostained cells and fibres were observed in the terminal tentacle, lateral lappets and the photosensory optic cushion, which is located at the base of the terminal tentacle (Fig. 6f, g).

Immunostained cells and/or fibres were revealed in the mucosa and basiepithelial nerve plexus, respectively, in many regions of the digestive system, including the peristomial membrane (Fig. 6b), oesophagus (Fig. 6h), cardiac stomach (Fig. 6i–k), pyloric stomach (Fig. 6l), pyloric duct (Fig. 6m), pyloric caeca (Fig. 6n) and intestine (Fig. 6o). The most intensely stained region of the digestive system was the cardiac stomach, with strongest staining in regions of the basiepithelial plexus adjacent to sites of attachment to the nodule and retractor strands, which were unstained (Fig. 6i).

Immunostained processes were also revealed in Tiedemann’s bodies (Fig. 6p), which are organs located in the ring canal (Fig. 2) that filter body fluids and contribute to generate the perivisceral coelomic fluids (Ferguson 1984). In the body wall, immunostaining was present in a nerve plexus located beneath the coelomic epithelium of the apical muscle and in the circular muscle layer (Fig. 6q). Immunostaining was observed in the basiepithelial nerve plexus of the external epithelium of the body wall both orally and aborally (Fig. 6r, s).

#### Immunohistochemical localization of ArLQP in *A. rubens*

ELISA analysis of serum from a rabbit immunized with an antigen peptide comprising the C-terminal region of the *A. rubens* luqin-type precursor (ArLQP) revealed the presence of antibodies to the antigen peptide (Fig. 3c). Immunohistochemical tests in which the ArLQP antiserum was tested alongside ArLQP antiserum pre-absorbed with the antigen peptide revealed specificity of immunostaining observed in the radial nerve cord (Fig. 4e, f). However, to optimise immunolabelling, affinity-purified antibodies (TEA eluate; 1:15 dilution) were used for detailed immunohistochemical analysis of ArLQP expression in *A. rubens*.

**Fig. 7:**
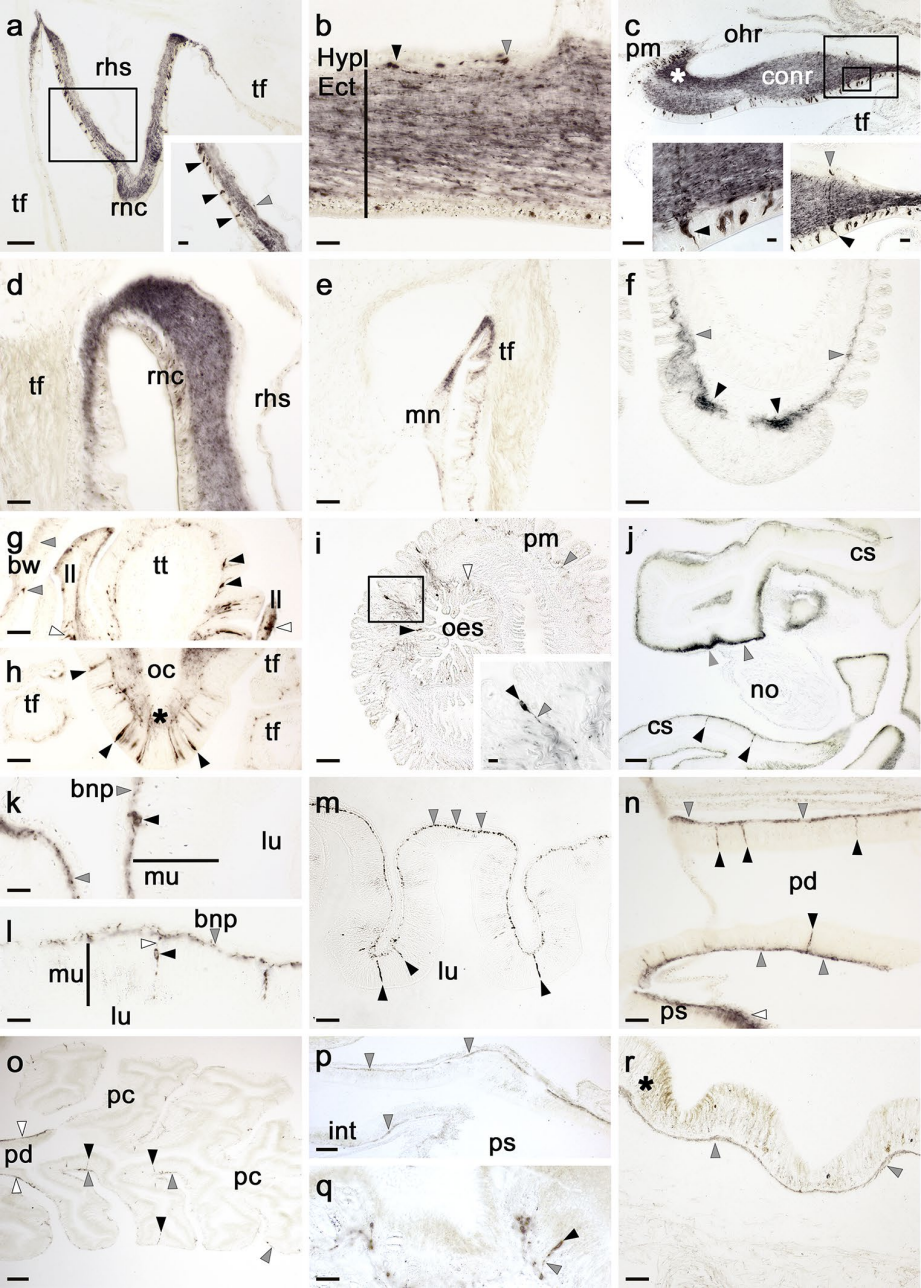
Immunohistochemical localisation of ArLQP in *Asterias rubens*. **a** ArLQP-immunoreactivity (ArLQP-ir) in a transverse section of the V-shaped radial nerve cord, with bilaterally symmetrical regional variation in the density of immunostaining in the ectoneural neuropile. The boxed region is shown at higher magnification in the inset, showing stained cell bodies in the hyponeural (grey arrowhead) and ectoneural (black arrowheads) regions of the radial nerve cord. **b** High magnification image of a longitudinal section of radial nerve cord showing an immunostained cell with a stained process in the hyponeural region (grey arrowhead) and an immunostained cell with a stained process in the ectoneural region (black arrowhead). Immunostained fibres can also be seen throughout the ectoneural neuropile. **c** ArLQP-ir in the circumoral nerve ring, showing regional variation in the intensity of immunostaining in the ectoneural neuropile and with a strongly stained region highlighted with an asterisk; the boxed regions are shown at higher magnification in the insets, where immunostained cells can be seen in the hyponeural region (grey arrowhead) and the ectoneural region (black arrowhead). **d** High magnification image of the junction between the radial nerve cord and an adjacent tube foot, showing ArLQP-ir in the ectoneural neuropile of the radial nerve cord and in the subepithelial nerve plexus of the adjacent tube foot. **e** ArLQP-ir in the marginal nerve and the subepithelial nerve plexus of an adjacent tube foot. **f** ArLQP-ir in the subepithelial nerve plexus (grey arrowheads) and basal nerve ring (black arrowheads) of a tube foot. **g** Horizontal section of an arm tip showing ArLQP-ir in cells/fibres located in the terminal tentacle (black arrowheads), a lateral lappet (white arrowheads) and the body wall surrounding the terminal tentacle (grey arrowheads). **h** Horizontal section of an arm tip showing ArLQP-ir in bipolar-shaped cells (black arrowheads) located in the photosensory epithelium and in the underlying neuropile (*) of the optic cushion. Immunostaining in adjacent tube feet can also be seen here. **i** Horizontal section of the central disc showing ArLQP-ir in the peristomial membrane and oesophagus. Immunostaining can be seen in the subepithelial nerve plexus of the peristomial membrane (grey arrowhead) and the oesophagus (white arrowhead). Immunostained cells can also be seen in the oesophagus (black arrowhead) and the peristomial membrane (boxed region). The inset shows the boxed region at higher magnification, with the cell body and process of an immunostained cell labelled with black and grey arrowheads, respectively. **j** ArLQP-ir in the lateral pouches of the cardiac stomach; note that the density of immunostained fibres is highest (grey arrowheads) in regions of the basiepithelial nerve plexus adjacent to the retractor strand and nodule, which are unstained. Immunostained bipolar-shaped cells (black arrowheads) can be seen in the mucosal layer of the cardiac stomach. **k**, **l** High magnification images of cardiac stomach tissue showing ArLQP-ir in cell bodies (black arrowheads) and processes in the basiepithelial nerve plexus (grey arrowheads); note that in **l** a process emanating from an immunostained cell body can be seen projecting into the plexus (white arrowhead). **m** ArLQP-ir in the pyloric stomach showing immunostaining in cells (black arrowheads) of the mucosa layer and processes in the basiepithelial nerve plexus (grey arrowhead). **n** ArLQP-ir in bipolar-shaped cells (black arrowheads) and in the basiepithelial nerve plexus (grey arrowheads) of the pyloric duct; intense immunostaining of the basiepithelial nerve plexus of the pyloric stomach can also be seen here (white arrowhead). **o** Horizontal section of an arm showing ArLQP-ir in the pyloric duct (white arrowheads) and in cells (black arrowheads) and the basiepithelial nerve plexus (grey arrowheads) of the pyloric caeca. **p** ArLQP-ir in the intestine, with immunostaining located in the basiepithelial nerve plexus (grey arrowheads). **q** ArLQP-ir in a rectal caecum, with an immunostained cell (black arrowhead) and a process emanating from it (grey arrowhead) highlighted. **r** ArLQP-ir in the basiepithelial nerve plexus (grey arrowhead) underlying the external epithelium of the body wall. A region of the external epithelium which is naturally pigmented and this is labelled with an asterisk to highlight that this is not ArLQP-ir. Abbreviations: bnp, basiepithelial nerve plexus; conr, circumoral nerve ring; cs, cardiac stomach; Ect, ectoneural; Hyp, hyponeural; ll, lateral lappet; mn, marginal nerve; mu, mucosa; nod, nodule; oc, optic cushion; oes, oesophagus; ohr, oral hemal ring; pm, peristomial membrane; pc, pyloric caecum; pd, pyloric duct; ps, pyloric stomach; rhs, radial hemal strand; rnc, radial nerve cord; tf, tube foot; tt, terminal tentacle. Scale bars: a, c, j, o, *p* = 60 μm; e, f, g, h, i, m, n, *r* = 32 μm; a-inset, b, c-right inset, d, k, l, *q* = 16 μm; c-left inset, i-insect = 6 μm

Antibodies to ArLQP revealed immunostaining in the major components of the nervous system of *A. rubens*, including the radial nerve cords, the circumoral nerve ring and the marginal nerves (Fig. 7a–e). In the radial nerve cord and circumoral nerve ring, immunostained cells were revealed in both the ectoneural and the hyponeural regions, but with far fewer stained cells in the hyponeural region than in the ectoneural region (Fig. 7a–c). Immunostained fibres were revealed throughout the ectoneural neuropile of the radial nerve cord and circumoral nerve ring, but with some bilaterally symmetrical regional variation in the density of immunostaining (Fig. 7a, c). Continuity of immunostaining can be seen between the ectoneural neuropile of the radial nerve cords and the subepithelial nerve plexus of a tube foot in the adjacent inner row of tube feet (Fig. 7a, d). Likewise, continuity of immunostaining can be seen between the marginal nerves and the subepithelial nerve plexus of a tube foot in the adjacent outer row of tube feet (Fig. 7e). In tube feet, immunostained fibres were revealed in the subepithelial nerve plexus along the length of the podium (Fig. 7a, e, f) and in the basal nerve ring of the disk region (Fig. 7f). In arm tips (Fig. 7g, h), immunostained cells and/or fibres were revealed in the terminal tentacle, optic cushion and lateral lappets.

Immunostained cells and/or fibres were revealed in the mucosa and basiepithelial nerve plexus, respectively, of many regions of the digestive system, including the peristomial membrane (Fig. 7i), oesophagus (Fig. 7i), cardiac stomach (Fig. 7j, k, l), pyloric stomach (Fig. 7m), pyloric duct (Fig. 7n, o), pyloric caeca (Fig. 7o), intestine (Fig. 7p) and rectal caeca (Fig. 7q). The strongest staining was observed in the lateral pouches of the cardiac stomach and in the pyloric duct. In the cardiac stomach, immunostaining was not evenly distributed throughout the folds of the cardiac stomach, with the most intense immunostaining located in the mucosal basiepithelial nerve plexus adjacent to the intrinsic retractor strands and nodule (Fig. 7j). Immunostaining was revealed in the basiepithelial nerve plexus on both the oral and aboral sides of the pyloric duct, with associated immunostained cells located in the mucosal layer (Fig. 7n). Immunostaining was observed in the basiepithelial nerve plexus of the diverticula of the pyloric caeca, but this was less extensive or intense than in the pyloric ducts (Fig. 7n, o). In the body wall, immunostaining was observed in the basiepithelial nerve plexus of the external epithelium (Fig. 7r).

## Discussion

Functional characterisation of neuropeptide signalling systems in the starfish *A. rubens* has been facilitated by the generation of antibodies to neuropeptides identified in this species and the use of these antibodies for immunohistochemical analysis of neuropeptide expression patterns. For example, detailed immunohistochemical analyses have been reported for pedal peptide type (Lin et al. 2017, 2018), gonadotropin-releasing hormone-type (Tian et al. 2017), calcitonin-type (Cai et al. 2018), vasopressin/oxytocin-type (Odekunle et al. 2019), somatostatin/allatostatin-C-type (Zhang et al. 2020, 2022), sulfakinin/cholecystokinin-type (Tinoco et al. 2021) and corticotropin-releasing hormone-type (Cai et al. 2021) neuropeptides in *A. rubens*. However, efforts to generate antibodies to some neuropeptides, notably the corazonin-type ArCRZ and the luqin-type neuropeptide ArLQ that are the focus of this study, have been unsuccessful. Therefore, here, we investigated an alternative approach by generating antibodies to peptide antigens corresponding to the C-terminal region of neuropeptide precursor proteins. The logic of this approach is that when neuropeptide precursors are subject to post-translational processing in the endoplasmic reticulum, the Golgi apparatus or secretory vesicles, cleavage of precursor proteins liberates neuropeptides but fragments of other non-bioactive parts of precursor proteins may also be retained with neuropeptides in secretory vesicles (Strand 1999). Therefore, immunohistochemical visualisation of non-neuropeptide peptides derived from precursor proteins may be reflective of the location of the neuropeptides. Furthermore, the C-terminal region of neuropeptide precursor proteins may also be detectable earlier than neuropeptides during neuropeptide precursor processing because neuropeptides are likely to only become detectable after cleavage at monobasic/dibasic sites and post-translational modifications (e.g. amidation, formation of disulphide bridges) have occurred, whereas the C-terminal region of the precursor protein is likely to be detectable immediately after protein synthesis has occurred in the endoplasmic reticulum.

### Assessment of neuropeptide precursor antibodies as markers for neuropeptide expression in *A. rubens* using the vasopressin/oxytocin-type precursor and neuropeptide (asterotocin) as a test case

With the aforementioned theoretical considerations in mind, here, we used the *A. rubens* vasopressin/oxytocin-type (asterotocin) precursor as a test case for comparison of immunostaining in *A. rubens* revealed by antibodies to a neuropeptide (asterotocin; (Odekunle et al. 2019)) and by antibodies to the C-terminal region of its precursor protein (ArASTP). This revealed extensive similarities in the patterns of immunostaining observed in *A. rubens* with the two antibody types, supportive of the prediction that antibodies to the C-terminal region of neuropeptide precursors can be used to visualise patterns of neuropeptide expression when production of antibodies to the bioactive neuropeptides has been unsuccessful. However, some differences in the patterns of immunostaining with the two antibody types were observed. For example, intense immunostaining was observed in the apical region of the radial nerve ectoneural neuropile with antibodies to the asterotocin precursor, whereas with asterotocin antibodies this region was largely void of immunostaining, consistent with previously reported findings (Odekunle et al. 2019). Likewise, asterotocin precursor antibodies, but not asterotocin antibodies, revealed immunostaining in the subepithelial nerve plexus of tube feet. How then can such differences in immunostaining be explained? One explanation would be differences in antibody titre and/or avidity and hence the intensity of staining, but an argument against this is our finding that patterns of immunostaining in many regions of the *A. rubens* body (e.g. in Fig. 5c, d showing the circumoral nerve ring) are strikingly similar both qualitatively and quantitatively. A second explanation would be that the asterotocin precursor antibodies are cross-reactive with another protein or proteins; however, our findings are inconsistent with this explanation in as much as the differences in the patterns of immunostaining observed with the two antibodies are not widespread, as might be expected, but instead are restricted to very specific regions of the nervous system. A third explanation is based upon our theoretical prediction that the C-terminal region of a neuropeptide precursor may be detectable with antibodies earlier in the biosynthetic pathway than the mature and post-translationally modified neuropeptide; for example, there are two post-translational modifications to the structure of asterotocin—C-terminal amidation and formation of a disulphide bridge between two cysteine residues. This temporal difference in the appearance of antigenicity in neurons may result in some spatial differences in patterns of immunostaing observed with neuropeptide-specific antibodies and non-neuropeptide but precursor-specific antibodies. Nevertheless, aside from the aforementioned minor differences in the patterns of immunostaining observed with antibodies to asterotocin and antibodies to the C-terminal region of the asterotocin precurors, it is clear from this test case that the use of antibodies to the C-terminal region of neuropeptide precursors is a useful approach for visualisation of neuropeptide expression when antibodies to bioactive neuropeptides cannot be produced. On this basis, we proceeded to investigate the expression of the corazonin-type neuropeptide ArCRZ and the luqin-type neuropeptide ArLQ, using antibodies to the C-terminal region of their corresponding precursor proteins.

### ArCRZ precursor immunoreactivity in *A. rubens*

When analysing the distribution of immunostaining revealed with antibodies to the C-terminal region of the ArCRZ precursor, we evaluated it by comparison with staining obtained using mRNA in situ hybridisation to localise transcripts encoding the ArCRZ precursor in *A. rubens* and reported previously (Tian et al. 2017). Although detection of neuropeptide precursor encoding transcripts in axons has been reported in other taxa (van Minnen and Bergman 2003), using our mRNA in situ hybridisation methods, we only observe staining in neuronal cell bodies in *A. rubens*. Therefore, comparison with immunostaining observed here with ArCRZP antibodies was limited to stained neuronal cell bodies. Nevertheless, as discussed below, the distribution of cells immunostained with the ArCRZ precursor antibodies, as reported here, was generally consistent with the distribution of ArCRZ precursor transcripts, as reported previously (Tian et al. 2017).

In the radial nerve cords and circumoral nerve ring, ArCRZ precursor transcripts are detected in cells located in the epithelium of the ectoneural region but not in the hyponeural region. Furthermore, ArCRZ precursor transcripts were also detected in the marginal nerves (Tian et al. 2017). Accordingly, antibodies to the ArCRZ precursor revealed stained cells in the epithelium of the ectoneural region of the radial nerve cords and circumoral nerve ring, but not in the hyponeural region, and in the marginal nerves. Furthermore, the ArCRZ precursor antibodies revealed immunostained processes of ectoneural cells, with regional variation in the density/intensity of immunostaining in the ectoneural neuropile. For example, the most intensely stained regions of the ectoneural neuropile are located laterally and apically, with the intervening region largely unstained. However, intensely stained varicose fibres can be seen traversing otherwise unstained neuropile in the medial aboral zone of the ectoneural region. Thus, the neuroarchitecture of ArCRZ precursor-expressing cells in the ectoneural region of the CNS in *A. rubens* has been revealed, extending previous findings based on the use of mRNA in situ hybridisation methods (Tian et al. 2017).

Turning to the body wall and associated appendages, ArCRZ precursor transcripts were revealed in cells located in the external epithelium of the body, the coelomic epithelial lining of the body wall overlying the apical muscle and proximal to the basal nerve ring in the disk region of tube feet (Tian et al. 2017). Here, the use of antibodies to the ArCRZ precursor revealed stained cells in the same locations, whilst also revealing stained fibres in these regions. Moreover, the visualisation of cells/processes expressing the ArCRZ precursor in the apical muscle and tube feet is consistent with previously reported in vitro pharmacological experiments, which revealed that ArCRZ causes contraction of apical muscle and tube foot preparations (Tian et al. 2017). Furthermore, in this study, we also extended analysis of ArCRZ precursor expression to the arm tip region of *A. rubens*, and here, immunostained cells and/or processes were revealed in sensory organs—the terminal tentacle and the associated optic cushion.

In the digestive system, mRNA in situ hybridisation revealed ArCRZ precursor transcripts in cells located in the mucosal layer of the cardiac stomach, pyloric stomach and pyloric duct (Tian et al. 2017). Accordingly, cells immunoreactive with antibodies to the ArCRZ precursor were revealed in the mucosa of these regions of the digestive system and also in the oesophagus, instestine and pyloric caeca. Furthermore, the ArCRZ precursor antibodies revealed immunostaining in the basiepithelial nerve plexus in all of these regions of the digestive system, with the most intense and extensive staining in regions of the cardiac stomach adjacent to sites of attachment to the nodule and retractor strands. In accordance with this abundance of immunostaining in the cardiac stomach, previous in vitro pharmacological studies have revealed that ArCRZ causes contraction of cardiac stomach preparations from *A. rubens* (Tian et al. 2017).

### ArLQ precursor immunoreactivity in *A. rubens*

As with the ArCRZ precursor, in analysing the distribution of immunostaining revealed with antibodies to the C-terminal region of the ArLQ precursor, we have evaluated it by comparison with staining obtained using mRNA in situ hybridisation to localise transcripts encoding the ArLQP precursor reported previously in *A. rubens* (Yañez-Guerra et al. 2018). The distribution of cells immunostained with the ArLQ precursor antibodies, as reported here, was generally consistent with the distribution of ArCRZ precursor transcripts, as reported previously (Yañez-Guerra et al. 2018). Furthermore, our immunohistochemical analysis enabled a more extensive anatomical investigation of ArLQ precursor expression in *A. rubens*.

In the radial nerve cords and circumoral nerve cords, antibodies to the ArLQ precursor revealed an extensive population of immunostained cells throughout the ectoneural epithelium, consistent with the distribution of cells containing ArLQ precursor transcripts revealed by mRNA in situ hybridisation methods (Yañez-Guerra et al. 2018). Accordingly, immunostained fibres were revealed throughout the ectoneural neuropile of the radial nerve cords and circumoral nerve ring, but with some regional variation in the density/intensity of immunostaining. When analysing ArLQ precursor expression in *A. rubens* using mRNA in situ hybridisation, we did not reveal transcripts in cells located in the hyponeural region of the radial nerve cords and circumoral nerve ring (Yañez-Guerra et al. 2018). Here, however, the use of antibodies to the ArLQ precursor revealed cells in the hyponeural region, although these were much less abundant than in the ectoneural region, and therefore the conflicting findings are probably explained by methodological differences in sensitivity and/or technical difficulty. Nevertheless, our discovery that ArLQ precursor expression is detected in hyponeural cells is noteworthy from a functional perspective because the hyponeural region of the CNS in starfish contains the cell bodies of skeletal motoneurons (Smith 1937; Mashanov et al. 2016). Therefore, we can infer that the ArLQ precursor is expressed by a small subpopulation of skeletal motoneurons, which contrasts with the other two neuropeptide precursors analysed in this study, ArASTP and ArCRZP, which do not appear to be expressed by hyponeural neurons.

Here, the use of ArLQ precursor antibodies revealed immunostained cells and/or processes in the body wall and associated organs in *A. rubens*. For example, the use of mRNA in situ hybridisation revealed ArLQ precursor transcripts located in cells located proximal to the basal nerve ring of the disk region in tube feet. Accordingly, here, extensive and intense immunostaining was revealed by ArLQ precursor antibodies in the basal nerve ring and in the subepithelial nerve plexus that extends along the length of tube foot podia. Consistent with this extensive immunostaining of the ArLQ precursor in tube feet, previous in vitro pharmacological studies have revealed that ArLQ causes dose-dependent relaxation of tube foot preparations (Yañez-Guerra et al. 2018). Furthermore, ArLQ is one of a number of neuropeptides that have been found to cause relaxation of tube foot preparations from *A. rubens* (Lin et al. 2017; Odekunle et al. 2019; Melarange and Elphick 2003; Cai et al. 2018; Zhang et al. 2020), which presumably reflects the complexity of the neurochemical mechanisms that control tube foot activity during starfish locomotion, feeding and other behaviours. In this study, we also extended analysis of ArLQ precursor expression to the arm tip region of *A. rubens*, and here, immunostained cells and/or processes were revealed in sensory organs—the terminal tentacle and the associated optic cushion. Therefore, the physiological roles of ArLQ are not restricted to the regulation of motor control but also, alongside other neuropeptides, are likely to be involved in neural mechanisms associated with the processing of sensory input to the CNS in starfish.

In the digestive system, mRNA in situ hybridisation revealed ArLQ precursor transcripts in cells located in the mucosal layer of the cardiac stomach and pyloric stomach (Yañez-Guerra et al. 2018). Accordingly, immunoreactive with antibodies to the ArLQ precursor was revealed in cells located in the mucosa of the cardiac stomach and pyloric stomach and in the basiepithelial nervous plexus of the cardiac stomach and pyloric stomach. Furthermore, visualisation of this pattern of expression was extended to other regions of the digestive system, including the peristomial membrane, oesophagus, intestine, pyloric duct and pyloric caeca. Interestingly, although extensive expression of the ArLQ precursor was observed in cardiac stomach of *A. rubens*, in vitro pharmacological tests with ArLQ have not revealed effects of ArLQ in the contractility of cardiac stomach preparations (Yañez-Guerra et al. 2018). Therefore, ArLQ may have an indirect modulatory role in regulation of cardiac stomach contractility and/or may be involved in regulation in other aspects of digestive system function such as ciliary mediated transport of food material or secretion of digestive enzymes.

## General conclusions

Previous studies have reported the use of antibodies to a non-neuropeptide fragment of neuropeptide precursor proteins for immunohistochemistry (Larsen et al. 1990; Larsen 1995). In this study, we report and validate the use of antibodies to the C-terminal region of neuropeptide precursors as a method for visualisation of neuropeptide expression patterns in the starfish *A. rubens*. The motivation for this technical approach came from several unsuccessful attempts to generate antibodies to neuropeptides, ArCRZ and ArLQ, derived from the *A. rubens* corazonin-type and luqin-type precursor proteins, respectively. We validated the technical approach by using the *A. rubens* vasopressin/oxytocin-type (asterotocin) precursor as a test case where antibodies to the neuropeptide had been successfully generated and used for immunohistochemistry. Then by generating antibodies to peptides corresponding to C-terminal regions of the ArCRZ and ArLQ precursor proteins, we have been able to use immunohistochemical methods to visualise for the first time the expression patterns of corazonin-type and luqin-type neuropeptides in starfish. This experimental approach could be employed for analysis of neuropeptide expression in other species in circumstances where the production of antibodies to bioactive neuropeptides has proven to be unsuccessful.

## Data Availability

Data supporting the findings of this study are available from the corresponding author, on request.
